# Assessing temporal dynamics of nitrogen surplus in Indian agriculture: district scale data from 1966 to 2017

**DOI:** 10.1038/s41597-024-04023-3

**Published:** 2024-11-02

**Authors:** Shekhar Sharan Goyal, Rohini Kumar, Udit Bhatia

**Affiliations:** 1 Department of Earth Sciences, Indian Institute of Technology Gandhinagar, Palaj, Gandhinagar, Gujarat 382055 India; 2 Computational Hydrosystems, Helmholtz Center for Environmental Research, UFZ, Leipzig, Germany; 3 Department of Civil Engineering, Indian Institute of Technology Gandhinagar, Palaj, Gandhinagar, Gujarat 382055 India; 4 Department of Computer Science & Engineering, Indian Institute of Technology Gandhinagar, Palaj, Gandhinagar, Gujarat 382055 India

**Keywords:** Environmental impact, Environmental impact

## Abstract

Nitrogen (N) is essential for agricultural productivity, yet its surplus poses significant environmental risks. Currently, over half of applied nitrogen is lost, resulting in resource wastage, contributing to increased greenhouse gas emissions and biodiversity loss. Excess nitrogen persists in the environment, contaminating soil and water bodies for decades. Quantifying detailed historical N-surplus estimation in India remains limited, despite national and global-scaled assessments. Our study develops a district-level dataset of annual agricultural N-surplus from 1966-2017, integrating 12 different estimates to address uncertainties arising from multiple data sources and methodological choices across major elements of the N surplus. This dataset supports flexible spatial aggregation, aiding policymakers in implementing effective nitrogen management strategies in India. In addition, we verified our estimates by comparing them with previous studies. This work underscores the importance of setting realistic nitrogen management targets that account for inherent uncertainties, paving the way for sustainable agricultural practices in India, reducing environmental impacts, and boosting productivity.

## Background & Summary

Nitrogen has been pivotal in sustaining nearly half of the global population^1–3^. With the advent of the Haber-Bosch process for Nitrogen Fixation (HBNF), the use of synthetic nitrogen fertilizers increased exponentially, doubling global grain production and supporting rapid population growth^1,4^. However, excessive application of nitrogen in agriculture has exceeded the safe planetary N boundaries^5^, resulting in significant environmental degradation, loss of biodiversity, and climate change^5–8^. Over 120 million tonnes of nitrogen fertilizer is annually applied to the global cropland, with more than half lost to the environment^9,10^. This surplus nitrogen has severely compromised the quality of surface and groundwater^11,12^, triggering harmful algal blooms^13^ and posing substantial risks to human health and aquatic ecosystems^14–16^. In response to these issues, the United Nations implemented a resolution focused on sustainable nitrogen management^17^, recognizing the need to minimize the harmful impacts of reactive nitrogen (Nr) and highlighting the importance of having long-term national and regional Nr data^18,19^. As the most populous nation in the world^20^, India has been at the forefront of this resolution, underscoring South Asia’s status as a significant hotspot for Nr pollution^21,22^. This context underscores the need for long-term N surplus data at finer granularity to inform decision-making and policy-making.

Indian agriculture has undergone significant transformations in the last century^22,23^. With its burgeoning population, the country’s agricultural demands have surged. Although India has achieved self-sufficiency in food production, it still accounts for 25% of the world’s undernourished population^24^. To meet this increasing food demand and sustain GDP growth, the reliance on synthetic fertilizers to improve agricultural productivity has increased markedly in recent decades^18^. This intensification of fertilizer application underscores the dual nature of reactive nitrogen (Nr), which can be a boon in the right place and a bane in the wrong, highlighting the need for careful analysis^25^. Consequently, the increased use of fertilizers has led to significant environmental impacts^26^, with varying degrees of severity in different regions of India^18,19,27,28^.

Nutrient-induced hypoxia (low oxygen levels) has been a global problem for decades, particularly in coastal regions, and is often attributed to the legacy effect of nitrogen^12,29,30^. Evaluating this impact requires long-term high-resolution nitrogen surplus data, which are sparse worldwide^10^. The N surplus, defined as the difference between nitrogen inputs (from fertilizer, manure, biologically fixed nitrogen, and atmospheric deposition) and nitrogen outputs (the nitrogen removed in harvested crop products), signifies potential nitrogen losses to the environment from agricultural soils^4,5,28,31,32^. Expressed in kg N ha^−1^ yr^−1^, this metric offers valuable insights into the environmental processes and sustainability of agricultural practices in India.

This study assesses the long-term nitrogen (N) surplus in the Indian agricultural system, a crucial indicator of potential environmental impacts. Specifically, we develop twelve long-term annual cropland nitrogen budget datasets (termed ‘N surplus’) for agricultural soil across India at the district level, covering more than half a century (1966–2017). This dataset explicitly accounts for uncertainties arising from input data sources and methodological choices in the major components of the N surplus, such as fertilizer, manure, and nitrogen removal rates. Our analysis underscores the importance of having a long-term dataset of N surplus, given the substantial changes in the magnitude of the N surplus over the past 50 years. The district-level database enables the aggregation of N surplus data at any spatial scale, making it relevant for the design of land and management strategies. Importantly, we validate the plausibility and consistency of our N surplus estimates by comparing them with existing N budget datasets^3,4,10^. Our detailed methodology, described below, provides a robust framework for exploring various assumptions in the estimation of the N surplus. This comprehensive data set would be instrumental in developing more precise and effective land management practices. In addition, it is highly relevant for water quality studies, as it can help identify regions at risk of N-induced hypoxia and groundwater contamination. Furthermore, the dataset can potentially support environmental impact assessments by linking nitrogen surplus to risks such as contaminated drinking water and related health issues and in surface waters with aquatic ecological risk indicators.

## Methods

**Fig. 1:**
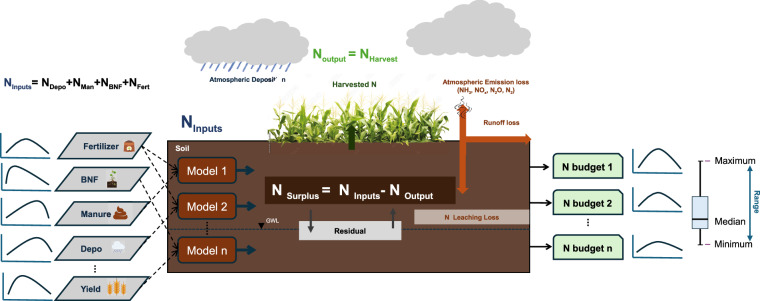
Conceptual figure of Nitrogen Budgets in Crop Production. This figure illustrates the nitrogen cycle in agricultural systems. Major nitrogen inputs, represented by blue arrows, include fertilizer, biological nitrogen fixation, manure, and atmospheric deposition. Nitrogen outputs, shown by the green arrow, are in the form of harvested crops. Nitrogen losses via leaching, runoff, and atmospheric emissions are depicted by orange arrows. Internal soil recycling processes are indicated by black arrows. The diagram also addresses uncertainties in nitrogen surplus calculations due to different methodological and data choices.

In this section, we outline our methodology for reconstructing a long-term annual time series of the various components that contribute to nitrogen (N) surplus at the district level from 1966 to 2017 (see Fig. 1 for a detailed workflow and data used). We compiled and standardized multiple databases that span different periods (1966-2017), varying frequencies (annual, decadal, and snapshots), and different spatial resolutions (basin-level, sub-basin-level, state-level, and district-level) using harmonization techniques from previous research^3,10,32,33^. The data sources used in this study are listed in Table 1. We developed 12 district-level N surplus estimates to comprehensively account for uncertainties arising from various methodological choices, data sources, and coefficients across key components of N surplus. Specifically, we combined one estimate for fertilizer use, three for animal manure, one for atmospheric deposition, two for biological nitrogen fixation, and two for nitrogen removal from cropland. This approach ensures that variability in data and methods is fully represented, offering a more robust and comprehensive understanding of N surplus at the district level.

**Table 1:** Datasets used in this study for N surplus calculation.

Datasets	Variables	Spatial Extent	Spatial Resolution	Temporal Extent	Temporal Resolution
ICRISAT^34^	Land use (cropland), Fertilizer application, Livestock, Crop harvested area and production	India	District	1996–2017	Annual
input4MIPS^36^	N atmospheric deposition	Global	1. 9° × 2. 5°	1850–2014	Monthly
Zhang *et al*.^42^	Animal manure application	Global	5 × 5	1860–2014	Annual
Lassaletta *et al*.^40^ and Bouwman *et al*.^49^	Crop nitrogen content, Biological fixation rate	Global	—	—	—
FAOSTAT^50^	Land use (cropland, pasture), fertilizer application, animal manure, crop harvested area, production and N content	Global	—	1961–2019	Annual
Rao, *et al*.^41^	Biological fixation rate	India	—	—	—
Regional literature^33,45–47^	% of Manure applied that is lost, burnt or used for other purposes	India	—	—	—

We used district-level data from the International Crops Research Institute for the Semi-Arid Tropics (ICRISAT)^34^, which provides an extensive record of various variables such as mineral fertilizer, livestock, crop production and crop harvested area within India from 1966 to 2017. ICRISAT offers comprehensive data for 314 districts in 20 states in India, covering the period from 1966-67 to 2017-18^35^. This dataset includes details on crop yield, net and gross area under cultivation, total synthetic nitrogen application, and livestock census. More information on the methodology used to calculate individual components of the nitrogen surplus is provided in the following subsections and in and Supplementary Information Fig. 1.

### Agricultural Cropland N Input

#### Atmospheric N Deposition

Nitrogen from the atmosphere, in both oxidized and reduced forms, is deposited on terrestrial and aquatic ecosystems through wet deposition (e.g. precipitation or snow) or through dry deposition (e.g. settling, impaction, and adsorption), a process collectively known as nitrogen deposition^36^. For this study, atmospheric nitrogen deposition, referred to as *N*_DEP_, was quantified using data from the Chemistry-Climate Model Initiative (CCMI) of the National Center for Atmospheric Research (NCAR). This dataset, which is part of the input datasets for Model Intercomparison Projects (input4MIPS)^36^, provides monthly time-step data from 1850 to 2014 at a spatial resolution of 1.9° latitude by 2.5° longitude; and had been used in previous N surplus reconstruction studies^32^. The data were interpolated to the required district resolution using nearest-neighbor interpolation and aggregated to an annual time step. To estimate nitrogen deposition for cropland, we multiplied the total nitrogen deposition by the proportion of the respective cropland area per district.

#### N Fertilizer

The amount of fertilizer applied to croplands is typically assessed using crop-specific synthetic nitrogen application rates. However, due to limited data availability, we used total nitrogen application data from ICRISAT^35^ (*N*_Total N fert applied_) applied to all agricultural crops. District-scale estimates of the total nitrogen fertilizer application rate (kgN ha^-1^ yr^-1^) in agriculture, referred to as *N*_FERT_, were obtained for the period 1966–2017, based on net sown area (also referred to as Net Cropped Area;NCA). In addition, gross nitrogen fertilizer rates (fertilizer applied in the total cropped area; GCA) were also obtained to understand the intensity of fertilizer application in the Indian agricultural system. Then we derived district-level total fertilizer application rates on net cropped area (kg N ha^−1^ yr^−1^) from 1966 to 2017 using Eq. (1).

1 $${{\rm{N}}}_{{\rm{FERT}}}(i,{y}_{1966-2017})=\frac{{N}_{{\rm{Total}}{\rm{N}}{\rm{fert}}{\rm{applied}}}(i,{y}_{1966-2017})}{{\rm{Net}}\,{\rm{cropped}}\,{\rm{area}}(i,{y}_{1966-2017})}$$

#### Biological N Fixation

Biological Nitrogen Fixation (BNF) refers to the microbial conversion of atmospheric nitrogen into a form usable by plants^37^. BNF is typically estimated using a crop-specific nitrogen fixation rate (for example, in units of kgN ha^-1^ yr^-1^)^38^ or as a linear function of crop yield^3,4,31,32,39^, using FAOSTAT yield data. These approaches can lead to variability due to differing regional and crop-specific parameterizations. In this analysis, the BNF for various cropland areas was calculated using both yield-based and area-based methodologies.

To estimate BNF for crops listed in the ICRISAT database, we used a yield-based method, considering yield as the primary indicator. This method accounts for factors such as crop type, soil and climate conditions, available nitrogen, soil moisture, crop vigor, and other management practices affecting N_2_ fixation, as shown in Eq. (2).

2 $${{\rm{N}}}_{{\rm{BNF}}}(i,{y}_{1966-2017})=\mathop{\sum }\limits_{c=1}^{nc}( \% {N}_{{{\rm{dfa}}}_{c}}\times {Y}_{c}\times {{\rm{NHI}}}_{c}\times {{\rm{BGN}}}_{c})$$

Here, nc is the total number of crop c in district i, %N_dfa_ represents the percentage of nitrogen uptake from fixation, *Y* is the crop-specific yield (in kg N ha^-1^ yr^-1^), NHI_c_ is the crop-specific nitrogen harvest index (the ratio of harvested material to total above-ground nitrogen production), and BGN_c_ accounts for total nitrogen fixation, including contributions from roots, nodules, rhizodeposition, decaying root cells and hyphae. These crop-specific rates are derived from previous studies^39,40^ and are mentioned in SI Table 2.

Further N fixation over cropland was also calculated using the area-based method (N_BNF_) which was derived by multiplying the respective crop area (*CA* (ha)) with their N fixation rates (*BNF*_*crops*−*Rate*_ (kg ha^-1^ yr^-1^))with nitrogen fixation rate taken from previous literature^41^ (SI Table 1), as summarized in Eq. (3).

3 $${{\rm{N}}}_{{\rm{BNF}}}(i,{y}_{1966-2017})=\mathop{\sum }\limits_{c=1}^{nc}\left(C{A}_{c}(i,c,{y}_{1966-2017})\right.\times BN{F}_{crops\mbox{--}Rate}$$

#### Manure N Inputs

Nitrogen added through manure is usually calculated by multiplying the estimated nitrogen excreted by livestock by the assumed fraction that is collected and applied to crops^42^. This method introduces significant uncertainties due to varying estimates of animal excretion, nitrogen content, manure management practices, and cropland distribution. Consequently, it represents one of the primary sources of uncertainty in nitrogen budgets^10^. In this study, nitrogen input from manure N_man_ was derived from waste excretion data associated with livestock production, utilizing two different livestock inventory data, one from the Indian Livestock Census from 1966 to 2017 and the other from the Global Livestock Impact Mapping System (GLIMS)^43^. Data from Indian Livestock Census taken from ICRISAT are provided at five-year intervals. Our analysis for this incorporated nine categories of livestock: cattle, buffalo, poultry, chickens, pigs, goats, and sheep, with data sourced from the State Department of Animal Husbandry and Dairying in India^44^. Using a combination of these data sources and methodologies, we have provided three different estimates for manure application in India to quantify the uncertainties involved.

Nitrogen excretion (total N manure production) from these animals was estimated by applying nitrogen excretion rates to livestock counts. In addition, the fate of the manure varies; it can be left in pasture or collected, stored, and then applied to agricultural soils. To accommodate these variabilities, we employ a methodology adapted from global studies (Lassaletta *et al*.^40^ and Zhang *et al*.^42^) and India-specific studies (Pathak *et al*.^33,45^) which assume that nitrogen excretion rates within a specific livestock category correlate with their slaughter weight. Consequently, national livestock-specific nitrogen excretion rates were taken from the literature^42^. These rates (*N*_rate(*j*)_) were then applied to the livestock counts to determine the total nitrogen excretion (*N*_man_) produced per district annually as shown in Eq. (4)

4 $${N}_{{\rm{ex}}(i,j)}={N}_{{\rm{rate}}(i,j)}\times \frac{{\rm{TAM}}(i,j)}{1000}\times 365$$

where *N*_ex(*i*, *j*)_ indicates annual N excretion for livestock category *j* from a specific district *i* for a particular year *y* (unit: kg N animal^−1^ yr^−1^), *N*_rate(*i*, *j*)_ indicates the default N excretion rate for livestock category *j* from a specific district *i* (unit: kg N (1000 kg animal mass)^−1^ day^−1^), and TAM(*i*, *j*) indicates Typical Animal Mass for livestock category *j* from a specific district *i* (unit: kg animal^−1^).

In addition, using livestock density data, we evaluated total manure nitrogen extreated by different livestock as shown in Eq. (5)

5 $${N}_{{\rm{man}}(i,j,y)}={N}_{{\rm{ex}}(i,j)}\times {D}_{(i,j,y)}$$

where *N*_man(*i*, *j*, *y*)_ indicates average nitrogen excretion per ha for livestock category *j* from a specific district *i* in year *y* (unit: kg N ha^−1^ yr^−1^). while *D* indicates the livestock density of animal *j* from a specific district *i* (unit: head ha^−1^ land in each district) in year *y*.

The proportion of N manure excreted that is finally used as applied to the croplands was calculated using two different approaches. Using methodology proposed by Zhang *et al*.^42^ we calculated livestock specific fraction of manure excretion application rate using the equation provided: Eq.(6)

6 $${F}_{M(j,{\rm{ProSys}})}={F}_{MT(j,{\rm{ProSys}})}\cdot (1-{F}_{MO(j,{\rm{ProSys}})})\cdot (1-{F}_{Loss(j,{\rm{ProSys}})})$$

where *F*_*M*(*j*, ProSys)_ indicates the fraction of manure from the livestock category *j* applied to cropland and *F*_*MT*(*j*, ProSys)_ indicates the fraction of total manure managed for different livestock production systems. *F*_*MO*(*j*, ProSys)_ indicates the fraction of managed manure that can be used for other purposes such as production of biogas. *F*_*Loss*(*j*, ProSys)_ indicates the fraction of managed manure lost through volatilization in the form of NH_3_ and NO_*x*_. All the parameters used in Eq. (6) can be found in previous literature^42^. ProSys indicates different livestock production systems that are irrigated and rainfed. Further total nitrogen applied through manure application was calculate using equation Eq.(7) mentioned below:

7 $${N}_{{\rm{manCR}}(i,y)}=\mathop{\sum }\limits_{j=1}^{{j}_{c}}{N}_{{\rm{man}}(i,j,y)}\times {F}_{M(j,{\rm{ProSys}})}$$

where *N*_manCR(*i*, *y*)_ indicates total manure nitrogen applied to the soils of the croplands for each district *i* in year *y*. Furthermore, combining the methodology proposed by Zhang *et al*.^42^ and the previous literature specific to the Indian region^33,45–47^ we converted the data of total manure production evaluated using Eq. (5) into manure applied to the cropland following Eq. (8)

8 $${N}_{{\rm{manCR}}(i,y)}=\mathop{\sum }\limits_{j=1}^{{j}_{c}}({N}_{{\rm{man}}(i,j,y)})\times [1-({{\rm{AM}}}_{{\rm{CN}}}+{{\rm{AM}}}_{{\rm{CL}}}+{{\rm{AM}}}_{{\rm{FL}}})]$$

where *j*_*c*_ represents the total number of livestock category (sheep, goat, buffalo, and cattle); ${{\rm{AM}}}_{{\rm{CN}}}$, AM_CL_, and AM_FL_ represent the fractions of animal manure, which is lost during collection, used as construction material, and burnt as fuel, respectively. The values of these fractions were taken from regional literature^33,45–47^.

To further compare and quantify uncertainty in our manure estimates, we utilized the gridded total manure application rate dataset provided by Zhang *et al*.^42^ as an additional estimate for manure application on agricultural cropland in India. This dataset was derived using the spatial distribution of livestock counts from the Global Livestock Impact Mapping System (GLIMS)^43^ and nitrogen excretion coefficients from the Intergovernmental Panel on Climate Change (IPCC)^48^, at a 5-arcminute spatial resolution, for the period 1966-2014. We assumed the constant manure application rates for the period from 2014 to 2017.

### Agricultural Cropland N output

#### Crop N Uptake

Harvested nitrogen (N) was determined by multiplying crop production by nitrogen content (kg N per kg of dry matter or wet weight of the harvested crop product). The total nitrogen uptake by crops (*N*_CROP_) was calculated using crop-specific production data at the district level obtained from the ICRISAT data archive (1966–2017). This calculation included 27 different crops, including fruits and vegetables, to estimate the nitrogen budget. Differences in nitrogen content parameters across various staple crops are the primary reason for the variations in harvested nitrogen content reported in different studies^3,40,49^. Although these variations are typically minor, they can result in significant discrepancies in national estimates of harvested nitrogen, given that a substantial portion of harvested nitrogen is derived from staple crops^10^.

To address this, we used nitrogen content data from two sources FAO^50^ and Bouwman *et al*.^49^. A uniform nitrogen content value was assumed for each crop or crop group, disregarding regional variations due to different crop varieties, management practices, yields, and temporal changes. This approach can introduce bias into the estimation of harvested nitrogen; however, quantifying this bias is difficult due to the scarcity of long-term and region-specific nitrogen content data. The parameter values used to convert the crop production data into the crop N uptake values (kg N ha^−1^ y^−1^) are provided in Tables S3 and S4. Eq. (9)

9 $${N}_{crop}(i,y)=\mathop{\sum }\limits_{c=1}^{{n}_{c}}\left({P}_{{r}_{{\rm{crops}}}}(i,c,{y}_{1850-2019})\times {N}_{{\rm{content}}}(c)\right)$$

where ${P}_{{r}_{{\rm{crops}}}}$ represents the crop-specific district-level production data for district *i*, crop *c*, and year *y*. Additionally, *N*_content_(*c*) represents the nitrogen content in crop *c*, and *n*_c_ represents the total number of crops in a district *i* for year *y*.

### Cropland N Surplus

We define the district-level N surplus, NS_dist_ (kg N ha^−1^ y^−1^), as the difference between the applied nitrogen inputs (fertiliser, manure, biological fixation, and atmospheric deposition) and “harvested” nitrogen outputs (crop N uptake)^3,31,40^ as discribed in Eq. (10).

10 $${{\rm{NS}}}_{{\rm{dist}}}={N}_{{\rm{DEP}}}+{N}_{{\rm{FERT}}}+{N}_{{\rm{BNF}}}+{N}_{{\rm{MAN}}}-{N}_{{\rm{CROP}}}$$

where *N*_DEP_ is atmospheric nitrogen deposition (kg N ha^−1^ y^−1^); *N*_FERT_ is N fertilizer (kg N ha^−1^ y^−1^); *N*_BNF_ is biological N fixation by crops (kg N ha^−1^ y^−1^); *N*_MAN_ is N in livestock waste applied to the cropland (kg N ha^−1^ y^−1^); *N*_CROP_ represents crop N uptake (kg N ha^−1^ y^−1^). For each district, the magnitudes are presented as the mean value of twelve nitrogen surplus dataNS_dist_ accompanied by their interquartile range (IQR).

### N surplus across India (1966-2017)

**Fig. 2:**
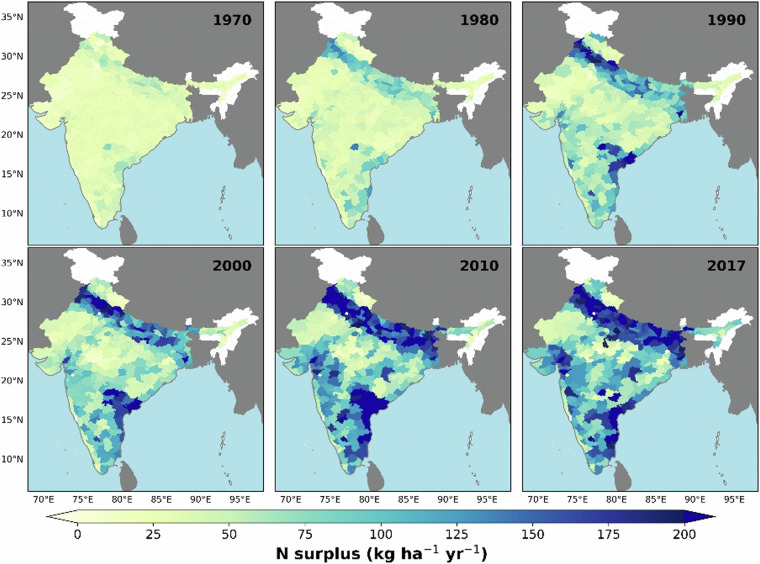
Spatial Distribution of Nitrogen Surplus in India. This figure presents snapshots of the nitrogen surplus (kg N ha^-1^ of agricultural cropland area yr^-1^) across India at district level, depicting the annual spatial variation in nitrogen surplus for the selected years(1970, 1980, 1990, 2000, 2010, 2017). The data shown represent the mean values derived from our twelve different nitrogen surplus estimates for each year. Regions in white indicate areas where data is unavailable.

**Fig. 3:**
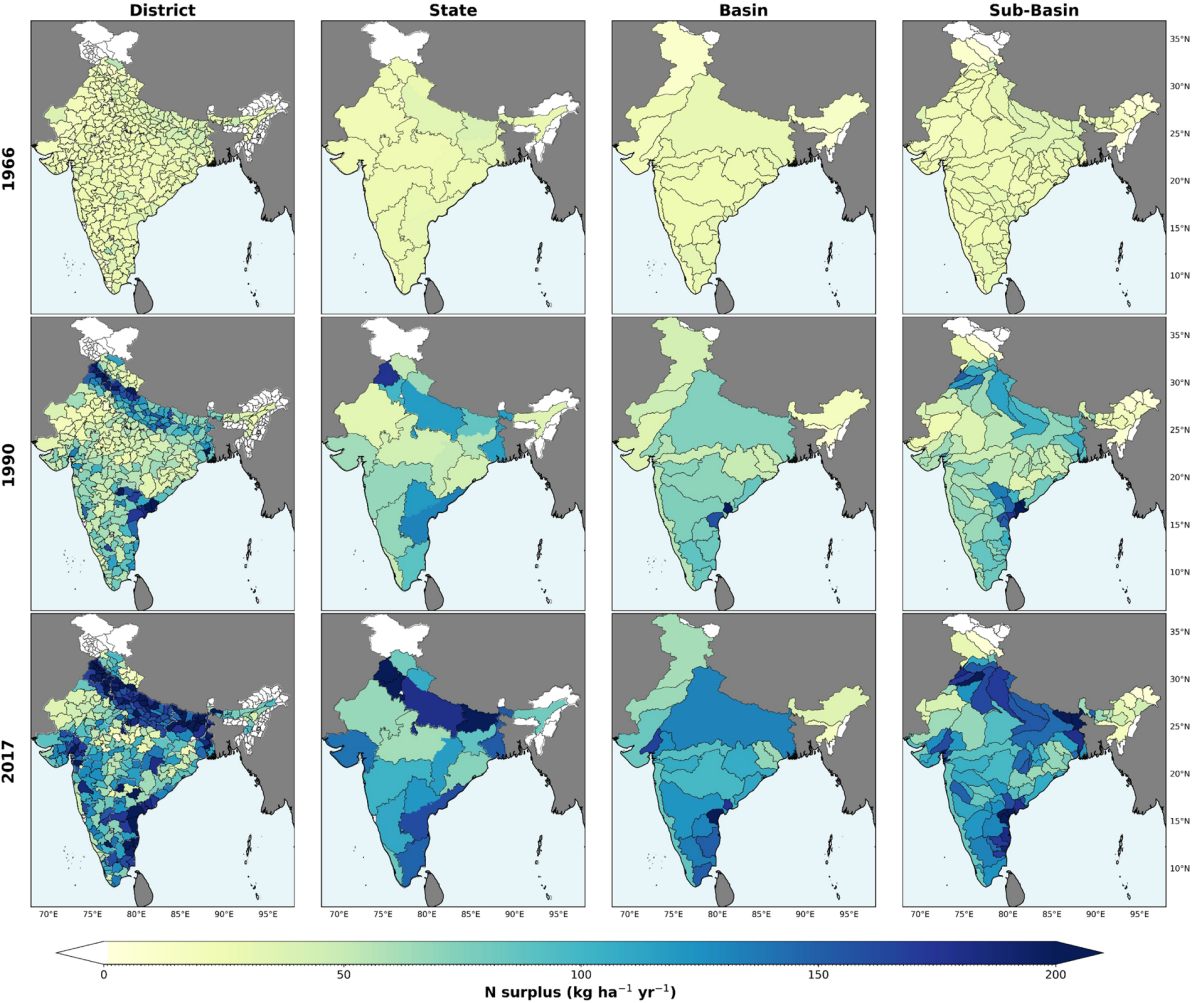
Nitrogen Surplus Across India at Various Spatio-Temporal Scale. This figure presents the total nitrogen surplus (kg ha^-1^ of agricultural cropland area yr^-1^) at different spatial scales for the years 1966, 1990, and 2017. The displayed values are the mean of twelve nitrogen surplus estimates. Regions in white indicate areas where data is unavailable.

**Fig. 4:**
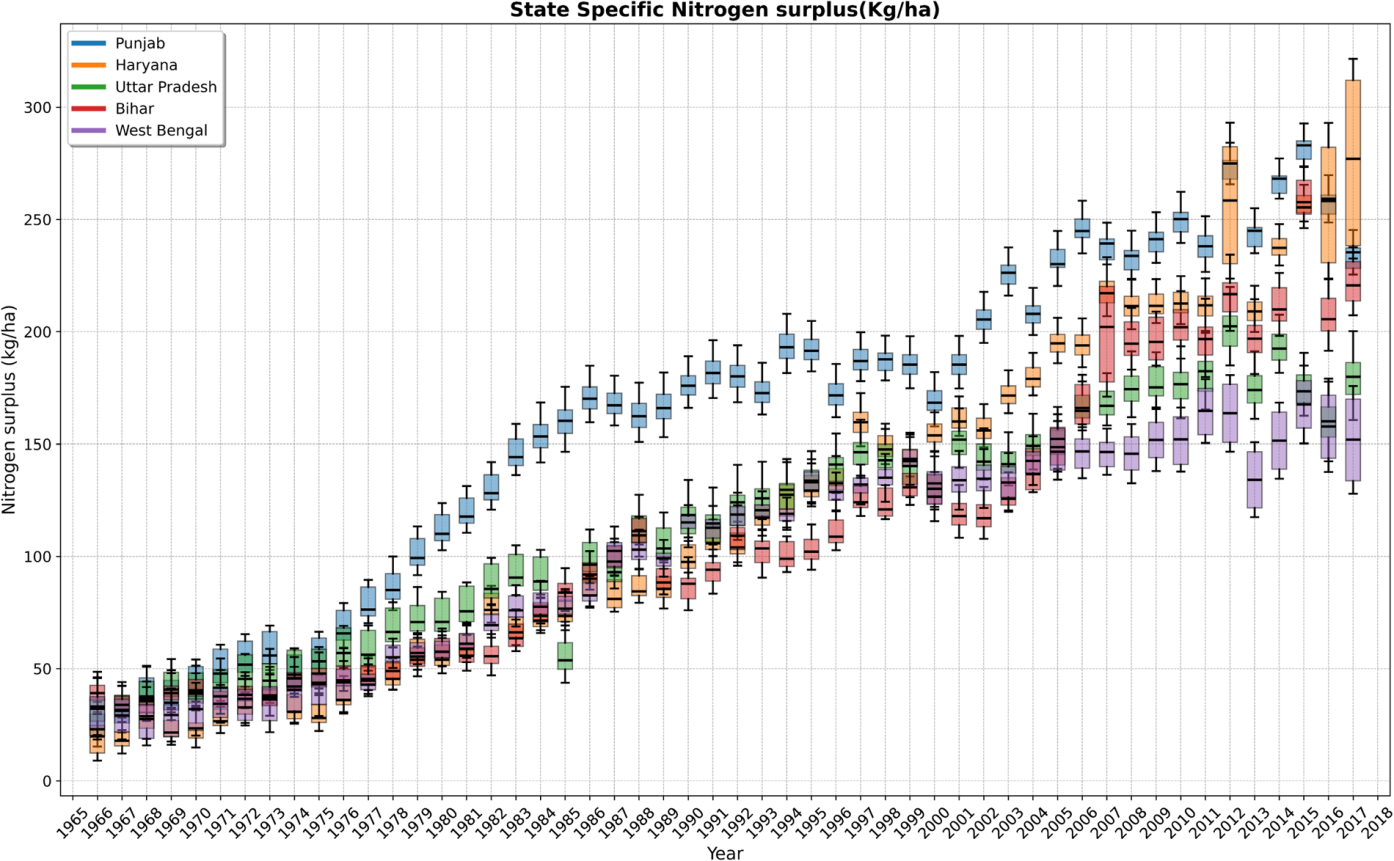
Temporal evolution of nitrogen surplus (kg ha^−1^ yr^−1^) across breadbasket regions of India, with uncertainties derived from twelve nitrogen surplus budget datasets from 1966 to 2017.

Then, we construct district-level nitrogen (N) surplus estimates across India for 1966 to 2017, incorporating uncertainties in the primary components of the N surplus. Our analysis used three data sets for animal manure, two for N removal, and two for biological fixation. Figures 2 and 3 display the mean values of these 12 N surplus estimates, while Fig. 4 illustrates the associated uncertainties involved at the state level. Further, Fig. 2 highlights the spatiotemporal variability of N surplus at the district level, with snapshots for the years 1970, 1980, 1990, 2000, 2010, and 2017. The district-level dataset in India enables us to evaluate nitrogen surplus at state, basin and sub-basin levels, as shown in Fig. 3. This figure shows the value of having information at state level, since country-level averaging of N surplus can conceal large disparities between the territories. We observed that breadbasket states such as Punjab, Haryana, Uttar Pradesh, Bihar, Telangana, Andhra Pradesh and West Bengal exhibited higher N surplus per unit net agricultural cropping area (kg N ha^−1^) compared to other Indian states, as visualized in SI Fig. 3. For instance, in 2000, the N surplus at country level for India took moderate values (89.1 kg N ha^−1^, respectively). However, state level information show that the N surplus was high in certain parts of India, with values reaching 168.9 kg N ha^−1^ in the Punjab and 154.7 kg N ha^−1^ in Haryana as shown in Fig. 4. Furthermore, significant temporal changes in nitrogen surplus were observed in India from 1966 to 2017. For example, in Punjab, nitrogen surplus increased from 24.4 kg N ha^−1^ in 1966 to 234.4 kg N ha^-1^ in 2017, and in Haryana, it rose from 19.4 to 276.4 kg N ha^−1^. Other states exhibited similar trends.

Further using basin level data provided by Central Water Commission(CWC)^51^ and level five hydro-shed basin classifications^52^, we aggregated nitrogen surplus data to analyze basin and sub-basin level variations across India as shown in Fig. 3. For instance, the Ganga River basin had a average nitrogen surplus of 74.3 kg N ha^−1^ in 1990, which increased to 132 kg N ha^−1^ in 2017. However, at the sub-basin level in 2017, the values varied significantly, ranging from 225.6 to 67.7 kg N ha^−1^, indicating greater variability at the sub-basin level. Additionally using the twelve nitrogen surplus estimates we quantified the uncertainties involved in surplus calculation in India. We found that recent years, such as 2017, showed the highest uncertainty. For example, in the state of Haryana, the nitrogen application was 276.4  ± 39.4 kg N ha^−1^. Overall we observed a varied level of uncertainty in N surplus across Indian states, emphasising the importance of accounting for the uncertainty due to methodological choices and dataset used to reconstruct N surplus^10,32^.

These findings highlight the importance of having a long-term dataset to quantify changes in nitrogen surplus over time at a finer spatial resolution. Further examining the spatiotemporal distribution of N surplus occurrences at the district level (SI Fig. 2) reveals that around 1966, India exhibited a higher probability for low N surplus values, predominantly peaking around 20-30 kg/ha. Only a limited number of districts exceeded this threshold, indicating limited fertilizer usage. However, in subsequent years, there has been a noticeable shift in the peaks of these distributions, reflecting a gradual increase in the N surplus generation, which can be attributed towards further increases in fertilizer application, and continued inefficiencies in N management^2,4^. In general, the changes from 1966 to 2017 highlight a trend of increasing N surplus over the decades, with peaks shifting to higher values and distributions exhibiting higher variance throughout India. This evolution suggests a change in agricultural practices that could lead to greater environmental impacts due to N runoff and leaching to receiving water bodies (groundwater or surface waters)^53^. Such trends underscore the dynamic nature of N management and its impacts over time. Our long-term annual datasets can facilitate understanding the extent and risk of nitrate-vulnerable regions across Indian cropland systems^27,54,55^ as well as to contrast Indian nitrate prone areas with other intensive cropping regions like in Europe^56,57^ or USA^29,58^. Furthermore, the flexibility of our dataset allows for aggregation and analysis at multiple scales, including basin, sub-basin, and district levels, which is crucial for developing regionally tailored policy interventions. While this study does not specifically address the most cost-effective scale for policy actions, future research could explore which spatial scale maximizes both policy impact and resource efficiency. This could offer valuable insights for more informed and effective decision-making processes.

## Data Records

The dataset^59^ consists of twelve different N surplus estimates, each stored in comma-separated value format (csv). These csv files feature an annual variable that quantifies N surplus in (kg N ha^-1^ yr^-1^) of the net cropped area per year, at the district level, covering 1966 to 2017. Additionally, SI Table 5 outlines the combination of data sources and methodologies used to generate each surplus estimate. We also offer N surplus estimates aggregated across various spatial scales—state, national, basin, and sub-basin levels obtained from CWC^51^ (see SI Table 6 for basin classification scheme used) and hydrosheds^52^ — encompassing regions throughout India. Detailed descriptions of the files included in the data repository^59^ are provided below: 10.5281/zenodo.13838035.

## Technical Validation

To evaluate the spatio-temporal plausibility and consistency of our N surplus dataset, we conducted a comparison with available datasets at the national level, given the absence of N surplus observations at finner resolution. We used data sets from Zhang *et al*.^4,10^ and Lüdemann *et al*.^3^, which provide data at the country level for India for the periods 1961-2015 and 1966-2018, respectively. The dataset from Lüdemann *et al*.^3^ serves as a reference, consistently delivering N land budgets for agricultural regions globally at the national level, while Zhang *et al*.^10^ synthesize information from 13 different N datasets to offer various components of the N surplus for croplands at the national level, positioning it as a benchmark for estimating uncertainty in national N surplus calculations.

**Fig. 5:**
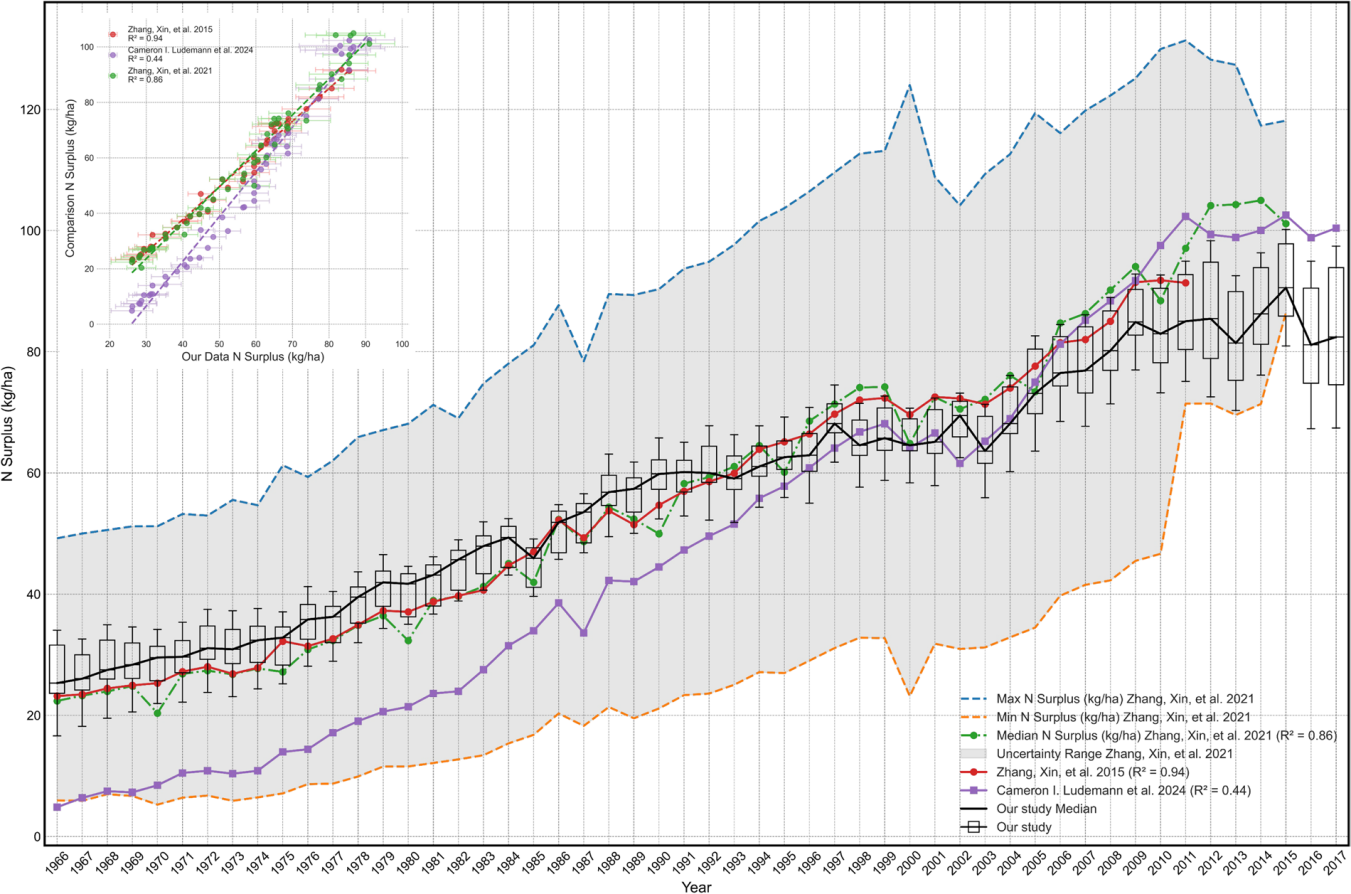
Comparison of N surplus (KgN/ha) in India from 1966–2017 between our study and previous literature^3,4,10^. The grey shaded area indicates the uncertainty bounds provided by Zhang *et al*.^10^, with median values shown in green. The purple line represents data from Lüdemann *et al*.^3^, and the red line represents values from Zhang *et al*.^4^. Data from our study is depicted as a box plot, illustrating uncertainties from 12 nitrogen surplus (KgN/ha) estimates. The panel in the upper left corner shows the linear fit between the mean of the 12 N surplus values for India over the years in the two studies: the x-axis shows the N surplus calculated in this study, and the y-axis presents the N surplus reported by Lüdemann *et al*.^3^ and Zhang *et al*.^4,10^. Bars indicate standard deviations, with the coefficient of determination (R^2^ value) mentioned in the legends.

We observed that the total fertilizer applied (kg) in Indian agricultural cropland matches both our data and that provided in previous literature^3,10^ (see SI Fig. 3). Furthermore, the gross fertilization rates (kg ha^−1^ yr^−1^) in our data align with those reported by previous researchers^3^. Therefore, we used the gross fertilizer application rate (total fertilizer applied divided by total cropping area) for data validation (Fig. 5), although it may underestimate the nitrogen surplus per unit cropland area in India. All analysis employed both net and gross rates, with Figs. 2, 3 and 4 reflecting net cropland area.

**Fig. 6:**
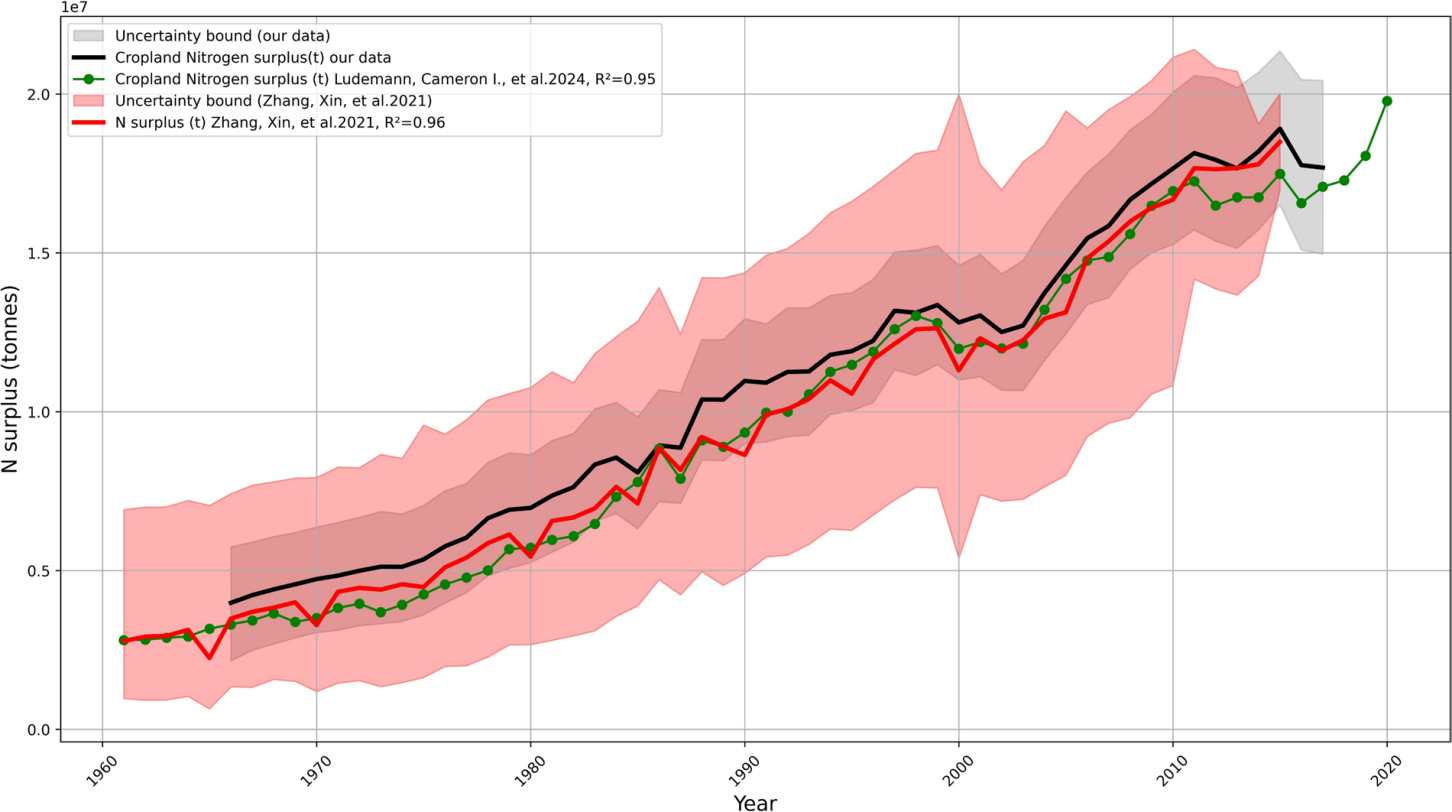
Comparison of total N surplus (tonnes) in India from 1966–2017 between this study and previous literature^3,10^. The red shaded area represents the uncertainty bound provided by Zhang *et al*.^10^, with the median value shown in red. Green represents values from Lüdemann *et al*.^3^. The grey shaded area represents uncertainties from 12 nitrogen surplus (tonnes) estimates, with the mean value represented by the black line. The goodness-of-fit R^2^ values between these datasets are mentioned in the legend.

In most cases, we could only extract data from previous literature at the country level, which we then used to compare with our national-level data. To evaluate the temporal consistency between our nitrogen surplus per unit cropland area (kg N ha^−1^ y^−1^) estimates and available datasets^3,10^, we calculated the coefficient of determination (*R*^2^). We report the mean and standard deviation of 12 *R*^2^ values, each derived from our 12 distinct nitrogen surplus datasets, as shown in Fig. 5. We observed a strong correlation between our nitrogen surplus per unit cropland area estimates and previously reported data. Specifically, our analysis yielded an *R*^2^ value of 0.94 when compared to the per unit cropland area nitrogen surplus (kg N ha^−1^ y^−1^) data reported by Zhang *et al*.^4^ at the national level. Additionally, we found an *R*^2^ value of 0.86 when compared to the data from Zhang *et al*.^10^. At the national level, the uncertainty intervals of our nitrogen surplus estimates (measured as one standard deviation around the mean of the 12 estimates) encompassed the values reported by Zhang *et al*.^10^. However, we observed a lower *R*^2^ value of 0.44 while comparing our estimates with N surplus per unit cropland area (kg N ha^−1^ y^−1^) reported by Ludemann *et al*.^3^. This discrepancy primarily arises from variations in the definitions of cropping areas, specifically the distinction between total cropping area and net sown area. To further assess the consistency of our data, we compared the total nitrogen surplus (Kg N) without normalizing by per hectare cropland area shown in Fig. 6. Our analysis revealed that our 12 national-level estimates for total nitrogen surplus (Kg N) aligned well with existing estimates, demonstrating similar spatio-temporal patterns and relative differences.

Specifically, our total agricultural cropland nitrogen surplus (kg N) for India from 1966 to 2014 is 512.16  ± 36.78 Tg N, while Zhang *et al*.^10^ reported a value of 504.56 Tg N. Furthermore, our dataset aligns well with the Total nitrogen surplus (kg N) values reported by Zhang *et al*.^10^, with an *R*^2^ value of 0.96, and Ludemann *et al*.^3^, with an *R*^2^ value of 0.95, as shown in Fig. 6. The observed differences in nitrogen surplus per unit agricultural cropland area highlight the necessity for a more detailed characterization of uncertainties in N surplus estimates to enhance their accuracy. It is important to note that N surplus datasets are not based on direct measurements but involve the use of inherently uncertain data and various methodological choices, each contributing to the overall uncertainty^10^.

## Usage Notes

This study provides annual estimates of nitrogen (N) surplus at the district level across India from 1966 to 2017^59^. We meticulously addressed uncertainties arising from various data sources and methodological choices, particularly those related to N inputs from biological fixation and manure, as well as N removal through crop harvesting, which are known to exhibit significant variability^10^. Our uncertainty ensemble successfully aligned with country-level N surplus estimates reported in previous studies and databases (see Section “Technical Validation”). While acknowledging that our dataset does not encompass all potential uncertainties, we have discussed additional data gaps and methodological considerations for future iterations of N surplus estimations (Section “Technical Validation”). Notably, we found that one of the uncertainties in our data arises from the definition of cropland area used. To ensure consistency with previous literature^3,10,50^, we have considered the Gross Cropland Area (GCA), which is defined as the total cropped area within a district. This approach acknowledges that the GCA can be greater than the Net Cropped Area (NCA), as it accounts for crop rotation and multiple cropping on the same land. Consequently, using the GCA may underestimate the fertilizer application rate within the field. To address this, we calculated the nitrogen (N) surplus (Kg/ha/y) using both GCA and NCA. Additionally, future efforts should prioritize the development of region-specific coefficients to further improve the accuracy of nitrogen surplus estimates. We recommend dedicated large-scale trials and experimental studies to gather data on key components, such as nitrogen removal, crop N content, and fertilizer application rates, under diverse agricultural and climatic conditions across India. Such an initiative would provide more precise and contextually relevant coefficients, allowing for a more granular understanding of nitrogen dynamics across the country. These efforts could facilitate the creation of a comprehensive national dataset, supporting improved nitrogen management strategies and more effective policy interventions tailored to regional needs.

## Supplementary information

Supplementary information

## Data Availability

We used the python for data processing. The codes for the study are available at https://github.com/shekharsg/N_surplus_data-
