# Supplementary material for: Assessing temporal dynamics of nitrogen surplus in Indian agriculture: district scale data from 1966 to 2017
Source: Sci Data. 2024 Nov 2;11:1191. doi: 10.1038/s41597-024-04023-3 (PMC11531528; doi:10.1038/s41597-024-04023-3)
Supplement: Supplementary file 1 — Supplementary information [file 41597_2024_4023_MOESM1_ESM.pdf]

Supplementary information

Assessing Temporal Dynamics of Nitrogen Surplus in Indian Agriculture: District-Scale Data from 1966 to 2017

Shekhar Sharan Goyal[1], *Rohini Kumar*[3*], *Udit Bhatia*[2*]

[1]Discipline of Earth Sciences, Indian Institute of Technology Gandhinagar, Palaj, Gandhinagar, 382055, Gujarat, India
[2]Discipline of Civil Engineering, Indian Institute of Technology Gandhinagar, Palaj, Gandhinagar, 382055, Gujarat, India
[3]Computational Hydrosystems, Helmholtz Center for Environmental Research, UFZ, Leipzig, Germany

[*]Correspondence to: rohini.kumar@ufz.de, bhatia.u@iitgn.ac.in

This file includes:

Supplementary Figures S1 to S3

Supplementary Table 1 to 4

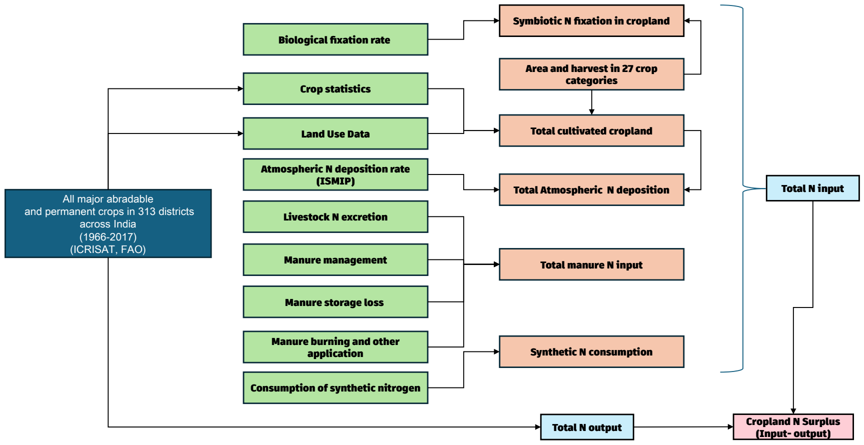

SI Fig. 1 – Workflow for constructing the long-term annual dataset of N surplus during the period 1966–2017

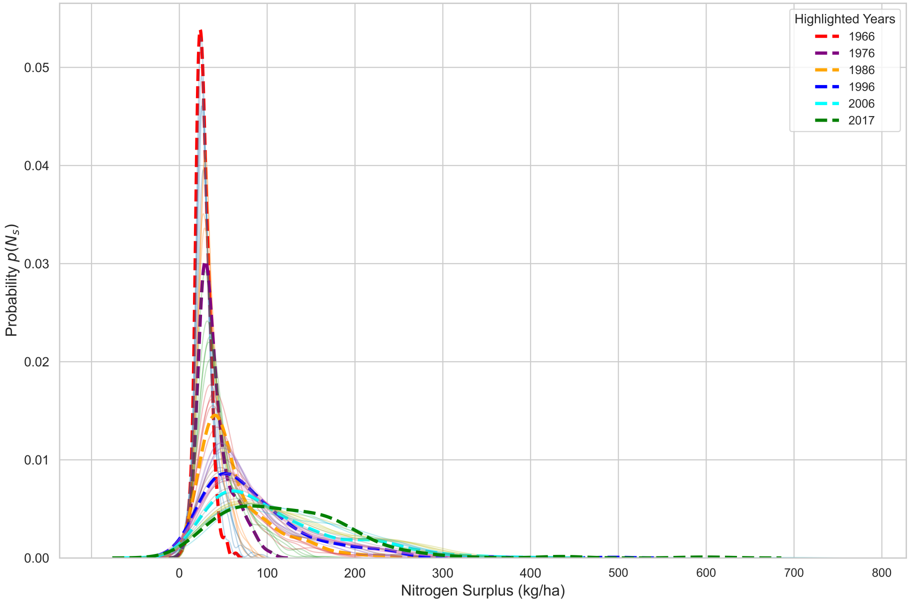

SI Fig. 2 – Empirical probability distribution of mean nitrogen surplus (KgN/ha) from 1966 to 2017 across India: Each year is represented by a unique color, with six year highlighted at decadal interval(1966, 1976, 1986, 1996, 2006, and 2017) to illustrate the temporal evolution of the nitrogen surplus distribution curve. The Y-axis represents the probability of occurrence of nitrogen surplus (KgN/ha) across 12 nitrogen budget models.

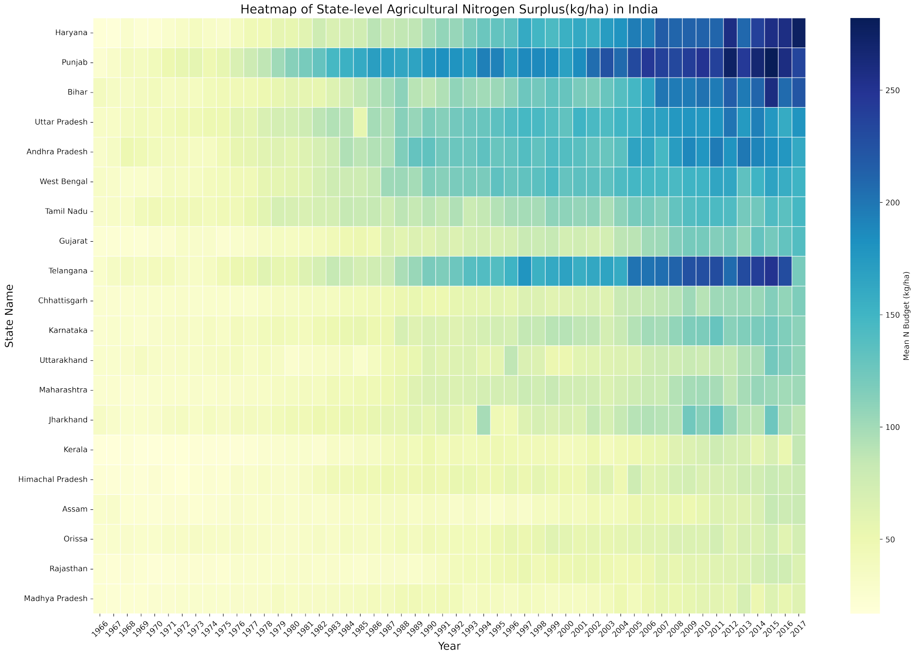

SI Fig. 3 – State specific Nitrogen Surplus in India: The heat map shows the mean nitrogen surplus (kg ha^{-1} yr^{-1}) across Indian states, derived from twelve nitrogen surplus budget datasets for net cropping area from 1966 to 2017. Rows represent states and columns represent years.

SI Table 1 – Biological nitrogen fixation rate taken from Rao, et al.[4]

No.	Crop	BNF rate (kg/ha)
0	Pigeon pea	60
1	Chick pea	60
2	Lentils	40
3	Groundnut	80
4	Soybean	80
5	Maize	13
6	Rice	22
7	Egyptian clover	150
8	Alfalfa	200
9	Wheat	13
10	Sugarcane	40
11	Oilseed	5
12	Cotton	5
13	Finger millet	5
14	Sorghum	5
15	Pearl millet	12
16	Oat	12
17	Barley	12
18	Makchari	12
19	Chara	12

SI Table 2 – Symbiotic N fixation rate taken from Lassaletta et al.[3]

Category	Legume	Location	% Ndfa	NHI	BGN
Fodder for forage, silage	Alfalfa, Clover etc.	Overall	78	0.90	1.7
Oilseeds	Groundnuts	Overall	68	0.50	1.3
Oilseeds	Soybean	Overall	57	0.73	1.4
Pulses	Common beans	Overall	40	0.75	1.3
Pulses	Faba bean	Overall	75	0.75	1.3
Pulses	Green leguminous	Overall	68	0.50	1.3
Pulses	Other pulses	Overall	68	0.75	1.3

SI Table 3 – N content for crops included in this study taken from [1] (g per kg)

**Crop**	**N content (g/kg)**
Wheat	19
Rice	13
Maize	14
Barley	17
Millet	15
Sorghum	15
Other cereals	16
Potatoes	3
Sweet potatoes	3
Cassava	2
Other root crops	3
Plantains	2
Sugar beets	2
Sugar cane	2
Pulses	35
Vegetables and melons	2
Bananas	2
Citrus fruit	1
Fruit excluding melons	1
Cocoa beans	14
Rapeseed	35
Oil palm fruit	15
Soybeans	35
Groundnuts in shell	40
Sunflower seed	34
Sesame seed	33
Other oilseeds	30
Coffee	24
Tea	78
Tobacco leaves	3
Seed cotton	29
Fibre crops primary	81

SI Table 4 – Nitrogen Removal Coefficients at standard moisture content for each crop (kg Nutrient removed per tonne crop produced) included in this study taken from [2]

**Crop**	**N Content (kg/tonne)**
BARLEY	18.0
CASTOR	14.4
CHICKPEA	27.6
COTTON	55.8
FINGER MILLET	20.4
FRUITS	1.4
FRUITS AND VEGETABLES	3.1
GROUNDNUT	33.6
KHARIF SORGHUM	14.6
LINSEED	28.8
MAIZE	12.4
OILSEEDS	13.1
ONION	2.6
PEARL MILLET	20.4
POTATOES	2.5
RABI SORGHUM	14.6
RAPESEED AND MUSTARD	31.4
RICE	12.9
SAFFLOWER	30.1
SORGHUM	14.6
SOYABEAN	59.3
SUGARCANE	4.7
SUNFLOWER	23.6
VEGETABLES	3.1
WHEAT	20.9

SI Table 5 – Combination of data and methodology used for N budget data preparation

**Budget**	**Synthetic Fertilizer Source**	**Manure Source**	**BNF Source**	**Nitrogen Deposition**	**N Removal Source**
N_budget_1_kg_ha	ICRISAT	Manure applied nitrogen using ICRISAT livestock data and Zhang et al. coefficients	BNF in agriculture	Using ISMIP	Nitrogen removal using coefficient by Bouwman et al. 2005
N_budget_2_kg_ha	ICRISAT	Manure applied nitrogen using coefficient from Pathak et al.	BNF in agriculture	Using ISMIP	Nitrogen removal using coefficient by Bouwman et al. 2005
N_budget_3_kg_ha	ICRISAT	Manure applied nitrogen using coefficient from Pathak et al.	BNF calculation using Lasestta et al	Using ISMIP	Nitrogen removal using coefficient by Bouwman et al. 2005
N_budget_4_kg_ha	ICRISAT	Manure applied nitrogen using ICRISAT livestock data and Zhang et al. coefficients	BNF calculation using Lasestta et al	Using ISMIP	Nitrogen removal using coefficient by Bouwman et al. 2005
N_budget_5_kg_ha	ICRISAT	Manure applied nitrogen using ICRISAT livestock data and Zhang et al. coefficients	BNF in agriculture	Using ISMIP	Nitrogen removal calculated using FAO nitrogen removal coefficient
N_budget_6_kg_ha	ICRISAT	Manure applied nitrogen using coefficient from Pathak et al.	BNF in agriculture	Using ISMIP	Nitrogen removal calculated using FAO nitrogen removal coefficient
N_budget_7_kg_ha	ICRISAT	Manure applied nitrogen using coefficient from Pathak et al.	BNF calculation using Lasestta et al	Using ISMIP	Nitrogen removal calculated using FAO nitrogen removal coefficient
N_budget_8_kg_ha	ICRISAT	Manure applied nitrogen using ICRISAT livestock data and Zhang et al. coefficients	BNF calculation using Lasestta et al	Using ISMIP	Nitrogen removal calculated using FAO nitrogen removal coefficient
N_budget_9_kg_ha	ICRISAT	Manure applied nitrogen calculated using Zhang et al. with GLIM data for livestock	BNF in agriculture	Using ISMIP	Nitrogen removal using coefficient by Bouwman et al. 2005
N_budget_10_kg_ha	ICRISAT	Manure applied nitrogen calculated using Zhang et al. with GLIM data for livestock	BNF calculation using Lasestta et al	Using ISMIP	Nitrogen removal using coefficient by Bouwman et al. 2005
N_budget_11_kg_ha	ICRISAT	Manure applied nitrogen calculated using Zhang et al. with GLIM data for livestock	BNF in agriculture	Using ISMIP	Nitrogen removal using coefficient by Bouwman et al. 2005
N_budget_12_kg_ha	ICRISAT	Manure applied nitrogen calculated using Zhang et al. with GLIM data for livestock	BNF calculation using Lasestta et al	Using ISMIP	Nitrogen removal calculated using FAO nitrogen removal coefficient

SI Table 6 – Basin Classification in India

Object ID	Basin Code	State	Basin Name	Latitude	Longitude
1	5	Tamil Nadu, Puducherry, Karnataka, Kerala	Cauvery	11.7413	77.4546
2	17	Andhra Pradesh, Karnataka, Tamil Nadu, Puducherry	East flowing rivers between Pennar and Kanyakumari	11.5634	78.7566
3	9	Andhra Pradesh, Karnataka	Pennar	14.4474	78.3375
4	3	Maharashtra, Telangana, Andhra Pradesh, Madhya Pradesh, Odisha, Karnataka, Puducherry, Chhattisgarh	Godavari	19.5135	78.8783
5	8	Madhya Pradesh, Chhattisgarh, Odisha, Jharkhand, Maharashtra	Mahanadi	21.208	83.1047
6	6	Odisha, Jharkhand, West Bengal	Subernarekha	22.4108	86.2949
7	7	Odisha, Jharkhand, Chhattisgarh	Brahmani and Baitarni	21.8358	85.3295
8	20	Manipur, Mizoram, Tripura, Nagaland	Minor rivers draining into Myanmar and Bangladesh	24.1224	93.6121
9	11	Gujarat, Rajasthan	Sabarmati	23.2465	72.6783
10	2C	Meghalaya, Manipur, Mizoram, Assam, Tripura, Nagaland	Barak and Others	24.5769	92.3871
11	2B	Arunachal Pradesh, Assam, Nagaland, Meghalaya, West Bengal, Sikkim	Brahmaputra	27.1532	93.2461
12	2A	Uttaranchal, Uttar Pradesh, Madhya Pradesh, Chhattisgarh, Bihar, Jharkhand, Rajasthan, West Bengal, Haryana, Himachal Pradesh, Union Territory of Delhi	Ganga	25.8641	80.863
13	1	Jammu and Kashmir, Himachal Pradesh, Punjab, Rajasthan, Haryana, Union Territory of Chandigarh	Indus (Up to border)	33.2054	75.9916
14	18	Rajasthan, Gujarat, Diu	West flowing rivers of Kutch and Saurashtra including Luni	24.0432	71.4987
15	12	Madhya Pradesh, Chhattisgarh, Gujarat, Maharashtra	Narmada	22.4319	77.3931
16	15	Karnataka, Kerala, Tamil Nadu, Puducherry	West flowing rivers from Tadri to Kanyakumari	11.0856	76.1274
17	19	Rajasthan	Area of Inland drainage in Rajasthan	27.8352	73.2857
18	4	Karnataka, Telangana, Andhra Pradesh, Maharashtra	Krishna	16.4734	76.5248
19		Jammu and Kashmir	Area of North Ladakh not draining into Indus Basin	35.4327	78.6346
20		Andaman and Nicobar Islands	Drainage Area of Andaman and Nicobar Islands Basin	11.1377	92.97
21		Lakshadweep Islands	Drainage Area of Lakshadweep Islands Basin	10.9342	73.0742
22	10	Rajasthan, Gujarat, Madhya Pradesh	Mahi	23.2229	74.014
23	13	Maharashtra, Madhya Pradesh, Gujarat	Tapi	21.0699	75.6779
24	14	Gujarat, Maharashtra, Dadara & Nagar Haveli, Goa, Daman	Diu, Karnataka & West flowing rivers from Tapi to Tadri	17.9307	73.6196
25	16	Andhra Pradesh, Odisha	East flowing rivers between Mahanadi and Pennar	18.7323	83.5857
26	16	Andhra Pradesh, Odisha	East flowing rivers between Mahanadi and Pennar	15.6348	79.6915
27	16	Andhra Pradesh, Odisha	East flowing rivers between Mahanadi and Pennar	16.781	81.1954
28	16	Andhra Pradesh, Odisha	East flowing rivers between Mahanadi and Pennar	16.308	81.312

Supplementary References

[1] AF Bouwman, G Van Drecht, and KW Van der Hoek. Surface n balances and reactive n loss to the environment from global intensive agricultural production systems for the period 1970–2030. *Science in China Series C: Life Sciences*, 48:767–779, 2005.

[2] Food and Agriculture Organization of the United Nations. *FAOSTAT Crops and Livestock Products*, 2022. Accessed: 2024-06-21.

[3] Luis Lassaletta, Gilles Billen, Bruna Grizzetti, Josette Garnier, Allison M Leach, and James N Galloway. Food and feed trade as a driver in the global nitrogen cycle: 50-year trends. *Biogeochemistry*, 118:225–241, 2014.

[4] DLN Rao and D Balachandar. Nitrogen inputs from biological nitrogen fixation in indian agriculture. In *The Indian nitrogen assessment*, pages 117–132. Elsevier, 2017.
